# Optical and Magneto-Optical Properties of Gd_22_Fe_78_ Thin Films in the Photon Energy Range From 1.5 to 5.5 eV

**DOI:** 10.3390/ma9010023

**Published:** 2016-01-02

**Authors:** Eva Jesenská, Takahiro Hashinaka, Takayuki Ishibashi, Lukáš Beran, Ján Dušek, Roman Antoš, Kiyoshi Kuga, Ken-ichi Aoshima, Kenji Machida, Hidekazu Kinjo, Martin Veis

**Affiliations:** 1 Department of Materials Science and Technology, Nagaoka University of Technology, Nagaoka, Niigata 1603-1, Japan; takahiro_hashinaka@mst.nagaokaut.ac.jp (T.H.); t_bashi@mst.nagaokaut.ac.jp (T.I.); 2 Institute of Physics, Charles University, Prague 12116, Czech Republic; beranlu@gmail.com (L.B.); r.jan.dusek@seznam.cz (J.D.); antos@karlov.mff.cuni.cz (R.A.); 3 Science and Technology Research Laboratories, Japan Broadcasting Corporation (NHK), Tokyo 157-8510, Japan; kuga.k-lu@nhk.or.jp (K.K.); aoshima.k-ia@nhk.or.jp (K.-i.A.); machida.k-ge@nhk.or.jp (K.M.); kinjou.h-lk@nhk.or.jp (H.K.)

**Keywords:** GdFe, magneto-optics, spectroscopic ellipsometry

## Abstract

Optical and magneto-optical properties of amorphous Gd_22_Fe_78_ (GdFe) thin films prepared by direct current (DC) sputtering on thermally oxidized substrates were characterized by the combination of spectroscopic ellipsometry and magneto-optical spectroscopy in the photon energy range from 1.5 to 5.5 eV. Thin SiN*_x_* and Ru coatings were used to prevent the GdFe surface oxidation and contamination. Using advanced theoretical models spectral dependence of the complete permittivity tensor and spectral dependence of the absorption coefficient were deduced from experimental data. No significant changes in the optical properties upon different coatings were observed, indicating reliability of used analysis.

## 1. Introduction

Considerable attention has been paid to magnetic and magneto-optical (MO) properties of amorphous ferrimagnetic thin films composed of rare-earth and transition metals because of their useful technological applications [1,2,3,4]. As one of the important magneto-optical storage materials, GdFe has significant advantages, such as large magnetization density, and possibility to adjust its compensation temperature, coercive and saturation magnetization by changing the composition [5,6,7]. Because of these properties, GdFe has a substantial impact on modern micro- and nanoelectronic research, where it is often used in domain wall junctions or MO memories [1,3,4].

The GdFe shows perpendicular anisotropy when the Fe concentration is about the compensation concentration, which is for this material about 75% [8]. This composition is often used for MO applications such as MO disk storage or MO spatial light modulator driven by spin transfer torque (spin spatial light modulator (SLM)) [2]. It is very important to control the GdFe composition precisely, since it significantly affects the GdFe magnetic switching property. Coercivity shows maxima when the composition is the compensation one, and it gets smaller when the composition becomes Fe rich (compared to the compensation composition). Spin-torque switching current of the spin-SLM is significantly reduced with an increase in Fe concentration and it shows very small switching current when composition is slightly Fe richer (such as Gd_20-22_Fe_78-80_) than the compensation one [9,10]. Therefore it is meaningful to investigate optical properties of the GdFe material with the Fe concentration around 78%.

The main purpose of our investigation was to fully determine the dielectric permittivity tensor of the GdFe thin film. Knowledge of the permittivity tensor is crucial, since it allows theoretical prediction of complex physical properties of complicated multilayered nanostructures containing GdFe layers without necessity to manufacture multiple samples. This is especially useful for the design of advanced devices as holographic 3D displays based on structures consisting of more than 10 nanolayers [2,11].

Since GdFe is very easy to oxidize [12], it is usually covered by a protecting layer, which complicates its analysis. The main reason is that the optical properties of protecting layer materials (here Ru, SiN*_x_*) may slightly differ in dependence on material they are deposited on. This is usually caused by the lattice mismatch between the film and the substrate, which induces strains of various kinds [13,14,15]. In this work, we have dealt with this problem by using two different coating layer materials which allowed more precise determination of GdFe permittivity tensor. Spectroscopic ellipsometry showed very similar optical properties of GdFe for both coatings, which allowed us to fit the optical constants from experimental data simultaneously.

We used spectroscopic ellipsometry at energies 1.2–6 eV and MO spectral measurements at energies 1.5–5.5 eV. From ellipsometric data we derived spectra of the real, ε_1*r*_, and imaginary, ε_1*i*_, part of diagonal permittivity tensor elements and the absorption coefficient spectra of the GdFe thin films. Magneto-optical properties were examined by polar magneto-optical Kerr effect (MOKE) rotation and ellipticity measurements. From these data we determined the spectral dependence of the real, ε_2*r*_, and imaginary, ε_2*i*_, part of off-diagonal GdFe permittivity tensor elements.

## 2. Results and Discussion

To obtain the GdFe dielectric permittivity tensor we analyzed optical and magneto-optical properties of two samples with structural compositions and nominal thicknesses listed in the Table 1.

**Table 1:** Structural compositions and nominal thicknesses of examined samples.

Sample	Substrate	Layer 1	Layer 2	Layer 3
Ru coated	Si	SiO_2_ (300 nm)	Gd_22_Fe_78_ (100 nm)	Ru (3 nm)
SiN*_x_* coated	Si	SiO_2_ (300 nm)	Gd_22_Fe_78_ (100 nm)	SiN*_x_* (20 nm)

### 2.1. Spectroscopic Ellipsometry

**Figure 1:**
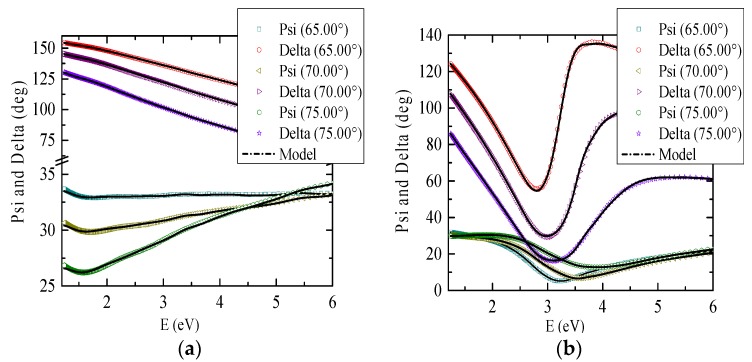
Measured variable angle spectroscopic ellipsometric data of (**a**) Ru coated sample and (**b**) SiN*_x_* coated sample are compared with theoretical calculations (lines). Dark cyan, olive and green symbols correspond to Psi measurements at angles 65°, 70°, 75° respectively. Red, purple and violet symbols correspond to Delta measurements at angles 65°, 70°, 75° respectively.

Figure 1 shows spectroscopic ellipsometry experimental data of studied samples. Since the measurements were performed under large incident angles, the difference between the ellipsometry data of two samples reflects not only the different optical parameters of coatings, but also the Fabry-Perot type resonance in much thicker SiN*_x_* layer with respect to Ru. The GdFe optical constants and layer thicknesses were fitted to the theoretical models (based on structural compositions) and experimental data. We used the “Multi Sample Analysis” mode to derive the GdFe optical constants from the experimental data of both samples simultaneously (It is an advanced mode in CompleteEase software that allows multiple samples to be fitted simultaneously with some of the fit parameters common to all samples (GdFe optical constants) and other allowed to vary (coating layer material, thicknesses) [16] (p. 169)). This could be done because the GdFe optical constants obtained from the individual sample analysis were very similar. Therefore this mode allowed, suppressing the fit error and so more precise analysis. Obtained optical functions were afterwards parameterized to ensure Kramers-Kronig consistent results. Optical functions of the GdFe thin film were parameterized by the linear summation of two Lorentz, two Gaussian oscillators and one Drude term in the spectral range 1.5–6 eV. Gaussian oscillators have been used due to their ability to rapidly approach zero beyond the FWHM positions which makes them suitable for the parametrization of the steeper regions of the optical spectra. Drude term has been used because GdFe is an amorphous alloy; therefore, we also had to consider the free carrier effect on the optical response. Parameters of used oscillators are listed in the Table 2. Derived thicknesses are listed in the Table 3. Optical constants of Si, SiO_2_, Ru and SiN*_x_* were determined from the ellipsometry measurements on individual samples. As we can see from Figure 1, the experimental and theoretical values correspond well. Since the GdFe has large absorption coefficient, the average penetration depth of this material in measured spectral region is around 24 nm, which allows consider the GdFe layer as semi-infinite. Therefore, we also theoretically modeled optical constants for structures where GdFe was used as semi-infinite substrate. However, no significant changes in results given by full and semi-infinite model structures were observed, since the full structure model also includes the high absorption of GdFe. In this work we present results obtained with the full model structure.

**Table 2:** Parameters of oscillators for model of GdFe layer for 1.5–6 eV spectral range. In here, *E* stands for central energies of oscillators; Amp represents amplitudes of oscillators and Br broadenings. For Drude term, *N* represents carrier concentration, μ carrier mobility and m* carrier effective mass.

Oscillator Type	*E* (eV)	Amp	Br (eV)
Lorentz	1.89	5.66	1.87
Lorentz	2.57	3.34	1.04
Gaussian	3.75	0.60	0.39
Gaussian	4.03	0.75	0.50
-	*N* (cm^−3^)	μ (cm^2^·V^−1^s^−1^)	m*
Drude	1.11 × 10^23^	0.36	0.53

**Table 3:** Thicknesses used to model GdFe layer for 1.5–6 eV spectral range. In here, *t* stands for thickness and *r* for roughness on top.

Sample	$t_{SiO_{2}}$ (nm)	*t*_GdFe_ (nm)	*t*_Ru_ (nm)	*t*_SiN_ (nm)	*r* (nm)
Ru coated	304	105	3.3	-	2
SiN*_x_* coated	304	105	-	21	3

**Figure 2:**
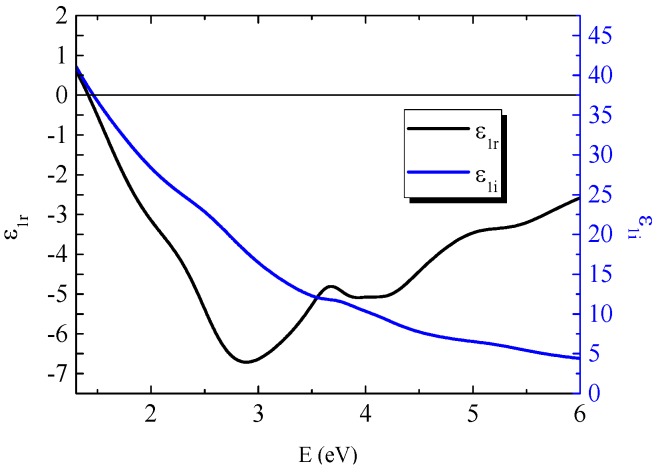
Real and imaginary part of diagonal permittivity tensor elements of GdFe. Black line corresponds to the real part ε_1*r*_ and blue line to the imaginary part ε_1*i*_ respectively.

**Figure 3:**
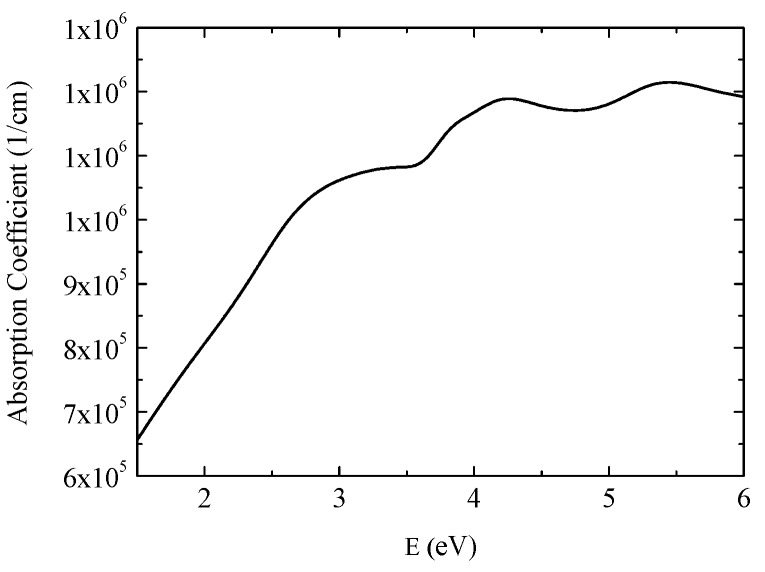
Calculated absorption coefficient of GdFe.

Figure 2 shows obtained spectra of the real, ε_1*r*_, and imaginary part, ε_1*i*_, of diagonal permittivity tensor elements. Figure 3 shows the calculated absorption coefficient spectra of the GdFe thin film. The ε_1*r*_ spectrum is characteristic by one global minimum at 2.9 eV while the ε_1*i*_ decreases its value with increasing energy in the whole measured spectral range. The spectra show similar behavior to Fe and Gd [17] (p. 394), [18] and also to previously reported results on GdFe films with different compositions [6,19]. The behavior in the spectral range 1.5–3 eV, where ε_1*r*_ decreases its value for higher energies is similar to the behavior of some transitions metals (including Cr, Ru, Ti, Gd [17] (p. 377), [20] (pp. 245, 259), [18]) and it was previously explained by intra-band transitions, which for some transition metals, are not negligible in a measured spectral region [17] (p. 375).

### 2.2. Magneto-Optical Kerr Effect *(*MOKE*)* Spectroscopy

**Figure 4:**
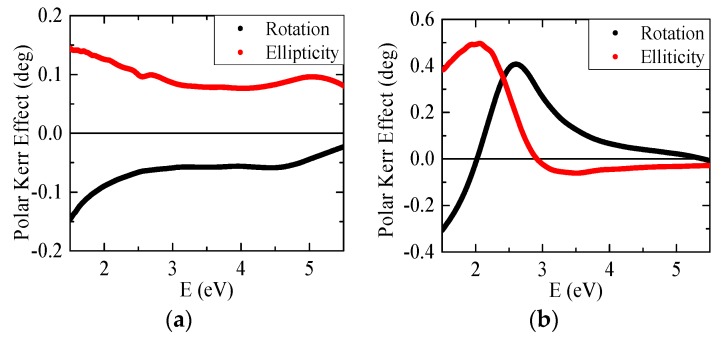
Polar magneto-optical Kerr effect (MOKE) rotation and ellipticity spectra of (**a**) Ru coated sample and (**b**) SiN*_x_* coated sample. Black symbols correspond to Kerr rotation; red symbols correspond to Kerr ellipticity.

Figure 4 shows experimental polar MOKE rotation and ellipticity spectra of the Ru and SiN*_x_* coated samples. The MOKE spectra of the Ru coated sample are characteristic by increasing rotation and ellipticity amplitudes toward to smaller energies. The rotation spectrum of the SiN*_x_* coated sample is characteristic by one global maximum at 2.6 eV and the ellipticity spectrum by one global maximum at 2 eV. We can also observe that the sample with SiN*_x_* coating is, especially in the spectral range 1.5–3.5 eV, giving much higher MO signal than the sample with Ru coating. This is probably caused by the multiple reflections inside SiN*_x_* coating layer, which results in Fabry-Perot like resonance causing the enhancement of the MOKE in this energy region. We used MOKE spectra to calculate the off-diagonal elements of the GdFe dielectric permittivity tensor.

**Figure 5:**
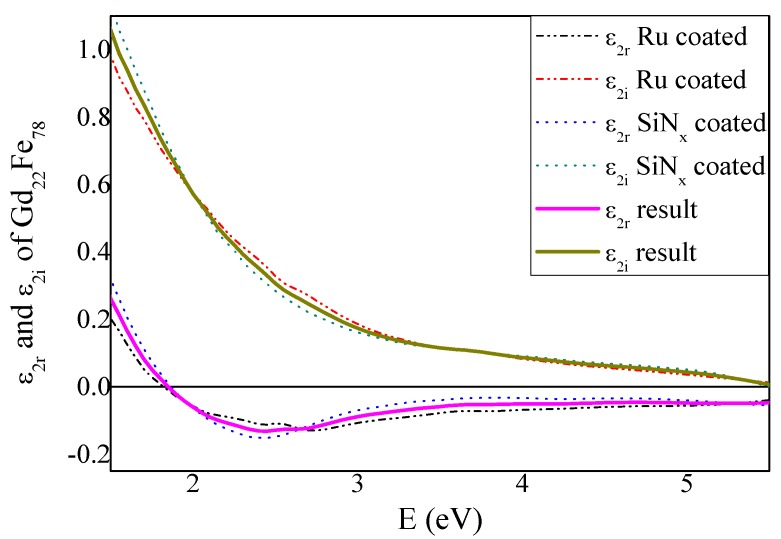
Real and imaginary part of off-diagonal permittivity tensor elements of GdFe. Dash-dot lines correspond to calculations from the Ru coated sample MOKE spectra, dotted lines correspond to calculations from the SiN*_x_* coated sample MOKE spectra. Pink line corresponds to the averaged real part ε_2r_ and green line to the averaged imaginary part ε_2*i*_ respectively.

For the off-diagonal elements calculations we used the diagonal elements of the GdFe dielectric permittivity tensor and thicknesses determined by the spectroscopic ellipsometry. The real and imaginary parts of off-diagonal elements, ε_2*r*_ and ε_2*i*_ were calculated from the MOKE spectra in the spectral range 1.5–5.5 eV. Figure 5 shows the spectra of the real and imaginary parts of off-diagonal permittivity tensor elements calculated from the MOKE spectra of the Ru and SiN*_x_* coated samples As one can see, the difference between the samples is rather small. Resulted values of the off-diagonal GdFe permittivity tensor elements ε_2*r*_ and ε_2*i*_ were obtained by averaging these two results and they are also shown in Figure 5. The ε_2*r*_ spectrum is characteristic by one global minimum around 2.5 eV while ε_2*i*_ is positive and decreases its amplitude with energy in the whole measured spectral range. The obtained spectra revealed a similar shape as off-diagonal permittivity elements of iron and also previously reported spectra of GdFe with different composition [19,21]. Amplitudes of GdFe off-diagonal permittivity elements spectra are smaller than amplitudes for Fe, which is most probably caused by the presence of the Gd.

## 3. Materials and Methods

### 3.1. Theory

The material response on electromagnetic waves in the presence of an external magnetic field can be described by the dielectric permittivity tensor. If the magnetization vector is parallel to the *z*-axis of the Cartesian coordinate system (the magnetic film-ambient interface is normal to the *z*-axis, light is propagating along the *z*-axis) and if we restrict ourselves to linear MO effects, the dielectric permittivity tensor simplifies to the form [22]:

(1) $$\left(\begin{array}{ccc} \varepsilon_{1} & -i\cdot \varepsilon_{2} & 0\\ i\cdot \varepsilon_{2} & \varepsilon_{1} & 0\\ 0 & 0 & \varepsilon_{1} \end{array}\right)$$

All elements of the tensor have real and imaginary parts:

(2) $$\begin{array}{c} \varepsilon_{1}=\varepsilon_{1r}-i\cdot \varepsilon_{1i}\\ \varepsilon_{2}=\varepsilon_{2r}-i\cdot \varepsilon_{2i} \end{array}$$

The optical behavior of the sample upon light reflection can be in Cartesian representation with the base of s and p polarizations described by the Jones matrix of reflection [23] (p. 170):

(3) $$J_{sp}^{R}=\left(\begin{array}{cc} r_{ss} & r_{sp}\\ r_{ps} & r_{pp} \end{array}\right)J_{sp}^{I}$$

Matrix elements are amplitude reflection coefficients for the *s* and *p* waves. The change in the polarization state of the reflected beam can be then expressed by the ellipsometric parameters Psi (ψ) and Delta (Δ), which are defined as:

(4) $$\tan\psi \cdot e^{i\Delta }=\rho =\frac{r_{pp}}{r_{ss}}$$

Where tanψ is the magnitude of the reflectivity ratio and Δ is the phase change between *s* and *p* polarized light. The *r_pp_* and *r*_ss_ are measured from the AC signal (dual rotating compensator ellipsometer configuration) [16] (p. 34). Analyzing the experimental ellipsometric data, one can derive the diagonal elements of the permittivity tensor. The important step in the spectroscopic ellipsometry analysis is the proper parametrization of the dispersion of the unknown optical functions. In this work we used Kramers-Kronig (KK) consistent Lorentz, Gaussian and Drude models. Classic version of Lorentz oscillator model can be mathematically described as:

(5) $$\varepsilon_{1\_Lorentz}=\frac{AmpBrE_{0}}{E_{0}^{2}-E^{2}-i\cdot EBr}$$

Parameters *E*_0_, Amp, Br denote the center energy, amplitude and the broadening parameter respectively [16] (p. 343), [24]. Gaussian line shape in ε_1*i*_ is defined as:

(6) $$\varepsilon_{1\_Gaussian}=Amp\left\{\begin{array}{l} \left(\Gamma \left(\frac{E-E_{0}}{\sigma }\right)+\Gamma \left(\frac{E+E_{0}}{\sigma }\right)\right)+\\ i\cdot \left(\text{exp}\left[-{\left(\frac{E-E_{0}}{\sigma }\right)}^{2}\right]+\text{exp}\left[-{\left(\frac{E+E_{0}}{\sigma }\right)}^{2}\right]\right) \end{array}\right\}$$

where

(7) $$\sigma =\frac{Br}{2\sqrt{\ln(2)}}$$

The function Γ is a convergence series that produces a Kramers-Kronig consistent line shape for ε_1*r*_ [16] (p. 344), [25]. In order to describe the free carrier effect on the dielectric response, Drude model is commonly used:

(8) $$\varepsilon_{1\_Drude}=\frac{-\hbar^{2}q^{2}N\mu }{\varepsilon_{0}(\mu m*m_{e}E^{2}+iq\hbar E)}$$

Parameters *N*, μ, m* denote the carrier concentration, carrier mobility and carrier effective mass respectively. The physical constants are ħ (Planck constant/2π), *q* (electron charge), ε_0_ (the vacuum dielectric constant) and *m*_e_ (the electron mass) [16] (p. 344) [26].

Spectroscopic MOKE can be used to derive off-diagonal elements of the permittivity tensor. In here we used the Yeh matrix formalism for anisotropic multilayers to theoretically calculate the MOKE effect in studied samples [23] (p. 344), [22,27]. The change in the polarization state of the reflected beam in the polar MOKE experiment can be expressed by the complex MO Kerr angle Φ_K_, which is for *p*-polarization and small angles of incidence defined as follows:

(9) $$\Phi_{K}=\theta_{K}-i\cdot e_{K}=\frac{r_{sp}}{r_{pp}}$$

In this equation θ*_k_* is the Kerr rotation, *e_k_* is the Kerr ellipticity. Let us consider the case of the three layered medium prepared on a bulk substrate. We will work in Cartesian coordinates where the sample interface is perpendicular to the *z*-axis, the wave vector of the incident light is perpendicular to the *x*-axis and each layer is characterized by the complex permittivity tensor and the thickness. In this case the Yeh Matrix Formalism allows to express the relationship between the electric field amplitudes on the substrate/film interface (*E*_0_^(0)^(*z*)) and the electric field amplitudes on the coating ambient interface (*E*_0_^(4)^(*z*_3_)) as:

(10) $$E_{0}^{\left(0\right)}(z)=\left\{\begin{array}{l} {\left[D^{\left(0\right)}\right]}^{-1}D^{\left(1\right)}P^{\left(1\right)}{\left[D^{\left(1\right)}\right]}^{-1}D^{\left(2\right)}P^{\left(2\right)}{\left[D^{\left(2\right)}\right]}^{-1}D^{\left(3\right)}\cdot \\ P^{\left(3\right)}{\left[D^{\left(3\right)}\right]}^{-1}D^{\left(4\right)}E_{0}^{\left(4\right)}(z_{3}) \end{array}\right\}=ME_{0}^{\left(4\right)}(z_{3})$$

Here *M* stands for transfer matrix (related to reflection coefficients) between substrate/film interface and coating ambient interface. Superscripts in brackets, *n* = 0, 1, 2, 3 and 4 are markers of the substrate (0), three layers and ambient half space (4). *P* stands for propagation matrix

(11) $$P_{ij}^{\left(n\right)}=\delta_{ij}\text{exp}(i\frac{\omega }{c}N_{zj}^{\left(n\right)}t_{n})$$

and *D* for dynamical matrix defined as:

(12) $$\begin{array}{l} D_{1j}^{\left(n\right)}=-\varepsilon_{2}^{\left(n\right)}(\varepsilon_{1}^{\left(n\right)}-N_{y}^{2})\\ D_{2j}^{\left(n\right)}=N_{zj}^{\left(n\right)}D_{1j}^{\left(n\right)}\\ D_{3j}^{\left(n\right)}=(\varepsilon_{1}^{\left(n\right)}-N_{y}^{2})(\varepsilon_{1}^{\left(n\right)}-N_{y}^{2}-{\left(N_{zj}^{\left(n\right)}\right)}^{2})\\ D_{4j}^{\left(n\right)}=-\varepsilon_{1}^{\left(n\right)}(\varepsilon_{1}^{\left(n\right)}-N_{y}^{2}-{\left(N_{zj}^{\left(n\right)}\right)}^{2}) \end{array}$$

where *t_n_*, *N_zj_* and *N_y_* are the thickness of the *n*-th layer, *z* components of the reduced wave vector and y components of the reduced wave vector respectively [23] (p. 151).

The structural composition and nominal thicknesses used for the theoretical analysis of ellipsometric and MOKE experimental data are described in the Table 1. The model structure consisted of Si semi-infinite substrate followed by 300 nm thick buffer layer of SiO_2_ and 100 nm thick layer of GdFe. Finally, it was followed by a coating which was 3 nm thick Ru layer for Ru coated sample and 20 nm thick SiN*_x_* layer in the case of SiN*_x_* coated sample. Surface roughness was also considered. We used the Bruggeman Effective Medium Approximation formula of the mixture of hosting material (ε) with void to simulate surface roughness as a thin layer with permittivity ε*_eff_* defined as follows [28]:

(13) $$0=(1-f)\frac{\varepsilon -\varepsilon_{eff}}{\varepsilon +2\varepsilon_{eff}}+\frac{1-\varepsilon_{eff}}{1+2\varepsilon_{eff}}$$

In this formula *f* denotes the volume fraction of the void in the mixture (we fixed this value to 50%).

### 3.2. Experimental Details

In this work we analyzed two samples with structural compositions and nominal thicknesses listed in the Table 1. The GdFe and Ru layers were deposited by direct current sputtering technique in Kr gas of pressure 8.7 × 10^−2^ Pa with a deposition rate of 3.6 nm/min. The SiN*_x_* film was prepared by RF ion beam sputtering technique in Kr gas of pressure 7.8 × 10^−2^ Pa with the deposition rate of 3.9 nm/min.

#### 3.2.1. Spectroscopic Ellipsometry Measurements

Spectroscopic Ellipsometry measurements were performed by a Mueller matrix ellipsometer Woollam RC2 (J.A. Woollam Co. Inc., Lincoln, NE, USA). We measured ellipsometric Psi and Delta parameters of the reflected light in the spectral range from 1.3 to 6 eV for incident angles 65°, 70° and 75°. Obtained experimental data were analyzed using CompleteEase software in “Multi Sample Analysis” mode.

#### 3.2.2. Magneto-Optical Measurements

Magneto-optical properties of samples were measured by the MOKE spectroscopy. The MOKE rotation and ellipticity spectra were measured in the polar configuration using a method of generalized magneto-optical ellipsometry with rotating analyzer, allowing the determination of the rotation angles with high accuracy. The spectra of polar Kerr rotation and ellipticity were acquired at the room temperature for nearly normal light incidence. Applied magnetic field was 1.2 T, which was enough for magnetic saturation of the samples. Incident light was p-polarized. The data were recorded in the photon energy range from 1.4 to 5.5 eV.

## 4. Conclusions

In this paper we presented systematic optical and magneto-optical study of the GdFe thin films prepared by DC sputtering technique. Since GdFe is easy to oxidize it was protected by the coating layers. Here Ru or SiN*_x_* layers were chosen. We used the assumption that optical and MO properties of the GdFe material will not change regardless of whether Ru or SiN*_x_* is deposited on its top. In experimental part, we performed spectroscopic ellipsometry and MOKE spectroscopy. Significant enhancement of MOKE was observed for the sample with SiN*_x_* coating, which was ascribed to the multiple reflections in SiN*_x_* layer. Combination of ellipsometric and MOKE spectroscopy allowed us to successfully determine the full dielectric permittivity tensor and the absorption coefficient spectra of the GdFe thin film prepared on the SiO_2_ buffer layer. Optical constants of Si, SiO_2_, Ru and SiN*_x_* used in our analysis were determined from the ellipsometry measurements on individual samples. The knowledge of the permittivity tensor is crucial, since it allows theoretical prediction of complex physical properties of complicated multilayered nanostructures containing GdFe layers without necessity to manufacture multiple samples.
